# Intravenous Sedation and Analgesia in a Pediatric Emergency Department: A Retrospective Descriptive Study

**DOI:** 10.7759/cureus.66451

**Published:** 2024-08-08

**Authors:** Madalena Carvalho, Ana Teresa Guerra, Marta Moniz, Carlos Escobar, Pedro Nunes, Vanda Bento, Clara Abadesso

**Affiliations:** 1 Pediatric Service, Child and Youth Department, Hospital Professor Doutor Fernando Fonseca, Lisboa, PRT

**Keywords:** analgesia, sedation, procedures, midazolam, ketamine, emergency

## Abstract

Background

Painful procedures in the pediatric emergency department often require the use of sedation and analgesia to ensure adequate pain control, a right of children and adolescents. This study aims to describe the procedural sedation and analgesia with intravenous medications performed in a pediatric emergency department.

Methods

This is a retrospective descriptive study of intravenous sedoanalgesia used in a pediatric emergency department of a level II district hospital in the Lisbon metropolitan area from October 2018 to December 2023. The type of intervention, drugs used, and adverse events were analyzed.

Results

A total of 615 patients were included in the study; 65.7% (n=404) were male with a median age of 6 years. The most frequently performed procedures were wound suturing (50.9%, n=313) and fracture reduction (36.3%, n=223). The drugs used for sedation and analgesia were ketamine (99.2%, n=610), midazolam (95.8%, n=589), propofol (1.6%, n=10), and morphine (0.5%, n=3). The majority of patients received midazolam and ketamine in association (93.8%, n=577). A total of 50 adverse events (8.1%) were recorded in 42 patients. The most frequent side effects were transient oxygen desaturation (2%, n=12), vomiting (1.5%, n=9), apnea/bradypnea (1%, n=6), and hallucinations (0.8%, n=5). The occurrence of adverse events was not dose-dependent (*p* >0.05). Respiratory complications resolved without requiring invasive interventions. Children were sedated by a pediatric intensivist in 68.1% (n=419), by a general pediatrician in 26.7% (n=164), and by a pediatric resident in 2% (n=12).

Conclusions

The results of this study demonstrate that intravenous sedoanalgesia, particularly the combination of ketamine and midazolam, is a safe method for sedation in pediatric patients, with a low rate of adverse events.

## Introduction

Diagnostic and therapeutic procedures are among the most frequent causes of pain, anxiety, and discomfort in children and adolescents accessing healthcare services. Small interventions can be painful, and the importance of adequate pain control is often underestimated [1,2]. Sedation and analgesia aim to control pain, reduce fear and anxiety, and control the child's movements during the procedures [1,3]. The choice of drugs or drug combinations should consider various factors to ensure adequate and safe sedation [4]. When performed properly, sedation/analgesia improves the quality and effectiveness of medical interventions [1]. Moreover, adequate relief of pain and anxiety inherent to painful procedures in children is an ethical imperative due to the short and long-term physical and psychological consequences of untreated pain [5]. It is the responsibility of pediatric healthcare providers to ensure optimal pain management in children [1,3,5].

Non-pharmacological methods, including a child/adolescent-friendly environment, age-appropriate distraction strategies, and the presence of parents are also essential for performing procedures [1,2]. There are many drugs available for pediatric procedural sedation with multiple possible routes of administration [6]. However, there is no consensus on the optimal drug or drug combination. The choice should consider patient characteristics (age, comorbidities) and the type and duration of the procedure [6].

The combination of sedative drugs (such as midazolam and propofol) with analgesic drugs (such as ketamine and fentanyl) is common [1]. The combination of midazolam and ketamine is widely used. Midazolam is a short-acting benzodiazepine with a rapid onset of action. It exhibits anxiolytic, amnestic, anticonvulsant, and muscle relaxant effects, without analgesic properties [4,7,8]. Respiratory depression can occur, and it is dose-dependent [4,8]. Ketamine has analgesic and amnestic properties, induces a dissociative effect, preserves airway protective reflexes, and has a minimal respiratory depression effect as well as cardiovascular stability [4,8,9]. Nausea and vomiting are common adverse reactions [4]. Disorientation, hallucinations, agitation, and nystagmus are also possible adverse effects [4,7]. Contrary to previous belief, the combination of midazolam with ketamine was not found to reduce adverse events such as hallucinations, agitation, and dysphoria [4,10,11]. On the other hand, it reduces the risk of vomiting but increases the risk of respiratory complications such as oxygen desaturation [4,11].

Sedation can have risks and younger children (less than 6 years old, particularly those under 6 months) may be at a higher risk of adverse events due to increased vulnerability to the effects of drugs on respiratory drive and airway patency [3]. Appropriate monitoring and evaluation are essential for the safety of the procedure [6]. Monitoring includes the surveillance of vital signs, including pulse oximetry and observation of the patient, particularly face and respiratory movements. It is crucial to rapidly detect adverse events such as respiratory depression, apnea, and airway obstruction [6,12].

This study aims to describe our experience with procedural sedoanalgesia using intravenous drugs in the pediatric emergency department (PED). The main drugs used in our emergency department are ketamine and midazolam.

## Materials and methods

This is a retrospective descriptive study. We included episodes in which intravenous sedation and analgesia were used in the pediatric emergency department (PED) of Hospital Prof. Doutor Fernando Fonseca (level II district hospital in the Lisbon metropolitan area) from October 2018 to December 2023 (5 years and 3 months). Patients aged between 0 and 18 years undergoing procedural sedation in the PED were included (the minimum age was 4 months, and the maximum age was 17 years).

In our department, we have a standardized protocol for procedural sedation and analgesia (PSA). Safety and monitoring guidelines are implemented. Monitoring during procedures with intravenous sedoanalgesia includes observation and cardiorespiratory monitoring (heart rate, respiratory rate, and peripheral oxygen saturation). The procedures are performed in a room with all the necessary equipment to properly manage serious adverse events. At least one physician and one nurse are present during the procedure.

To conduct this study, we used an anonymized database completed by the physician responsible for intravenous sedation. Physicians responsible for completing the database included pediatric intensivists, general pediatricians, or pediatric residents (under the supervision of a pediatrician). The database consisted of a short questionnaire that collected the following data: age, gender, type of procedure, drugs and doses used, duration of sedation, adverse events, and physician responsible for sedation (pediatric intensivist, general pediatrician, or pediatric resident). Patients receiving sedative and analgesic drugs by non-intravenous routes were excluded.

For this study, adverse events were defined as follows: hypoxemia (peripheral oxygen saturation <92%), vomiting, apnea/bradypnea, secretions/sialorrhea, agitation, hiccups, cough, partial upper airway obstruction, urinary incontinence, and urticaria. The research team defined adverse events based on their collective expertise. Severe adverse events include respiratory complications requiring ventilation, complete airway obstruction, and significant hypotension or bradycardia requiring intervention. During the procedure, all patients were monitored; however, periodic systematic recording of vital signs was not performed. Nonpharmacologic support for sedoanalgesia was employed. Statistical analysis was performed using Microsoft Excel^®^ (Microsoft Corporation, Redmond, WA) and IBM® SPSS® Statistics version 28 (independent t-test and chi-square, significance level set at p<0.05; SPSS V28, Armonk, NY, US). The study was approved by the hospital's ethics committee.

## Results

During the study period, 615 intravenous sedations were performed in the PED. Of the patients 65.7% (n=404) were male. The median age was 6 years (minimum 4 months, maximum 17 years). Intravenous sedoanalgesia was used in painful procedures and/or procedures where it was necessary that the patient remain quiet (Table 1). The most common procedures were wound suturing (50.9%, n=313), fracture reduction (36.3%, n=223), and lumbar puncture (4.7%, n=29). The median duration of the procedures was 10 minutes (minimum 1 minute, maximum 130 minutes), recorded in 540 patients.

**Table 1:** Procedures performed with sedation and analgesia

Procedures	% (n)
Wound suturing	50.9% (313)
Fracture reduction	36.3% (223)
Lumbar puncture	4.7% (29)
Abscess drainage	3.6% (22)
Foreign body removal	2.3% (14)
Complementary diagnostic exams	1% (6)
Wound cleaning/ dressings	0.8% (5)
Reduction of paraphimosis	0.3% (2)
Joint aspiration	0.2% (1)

The drugs used were ketamine (99.2%, n=610), midazolam (95.8%, n=589), propofol (1.6%, n=10), and morphine (0.5%, n=3). The drugs used are described in Table 2.

**Table 2:** Drugs used in intravenous sedation and analgesia

Drugs	% (n)
Midazolam and Ketamine	93.8% (577)
Ketamine	3.4% (21)
Midazolam, Ketamine, and Propofol	0.8% (5)
Midazolam	0.7% (4)
Propofol and Ketamine	0.7% (4)
Midazolam, Ketamine and Morphine	0.5% (3)
Propofol	0.2% (1)

The combination of ketamine and midazolam was used in 93.8% of procedures (n=577). In the group of patients who underwent this combination, the majority (62.6%, n=361) received a dose of 0.1 mg/kg midazolam and 1 mg/kg ketamine. In this group, 33.4% of patients (n=193) required a higher number of boluses of at least one of the drugs to achieve adequate sedation. In 4.2% (n=26) of patients, monotherapy was performed: ketamine (3.4%, n=21), midazolam (0.7%, n=4), and propofol (0.2%, n=1). The remaining patients had the following combinations: midazolam/ketamine/propofol (0.8%, n=5), propofol/ketamine (0.7%, n=4), and midazolam/ketamine/morphine (0.5%, n=3). The average dose of midazolam used was 0.106 mg/kg (minimum 0.03 mg/kg; maximum cumulative dose 0.3 mg/kg), and the average dose of ketamine was 1.3 mg/kg (minimum 0.5 mg/kg; maximum cumulative dose 7 mg/kg).

Fifty adverse events (8.1%) were recorded in 42 patients. The most frequent were transient oxygen desaturation (2%, n=12), vomiting (1.5%, n=9), apnea/bradypnea (1%, n=6), and hallucinations (0.8%, n=5). The adverse events are described in Table 3. Respiratory complications were the most frequent (3.9%, n=24). All of them resolved with positioning measures and supplemental oxygen. Invasive airway interventions were not required. Serious adverse events did not occur.

**Table 3:** Adverse events

Adverse effects	% (n)
Transient oxygen desaturation	2% (12)
Vomiting	1.5% (9)
Apnea/bradypnea	1% (6)
Hallucinations	0.8% (5)
Hiccups	0.7% (4)
Cough	0.7% (4)
Secretions/sialorrhea	0.5% (3)
Agitation	0.5% (3)
Partial obstruction of the upper airway	0.3% (2)
Urinary incontinence	0.2% (1)
Urticaria	0.2% (1)

The age groups with a higher relative percentage of adverse events were infancy (18.2%) and adolescence (11.7%) as seen in Table 4.

**Table 4:** Distribution of adverse events by age groups

Age groups		Adverse events		Total
		Yes	No	
Infancy (0-12 months)	Count (n)	2	9	11
	% within age group	18.2%	81.8%	100%
Toddler (13-24 months)	Count (n)	2	51	53
	% within age group	3.8%	96.2%	100%
Early Childhood (2-5 years)	Count (n)	10	233	243
	% within age group	4.1%	95.9%	100%
Middle Childhood (6-11 years)	Count (n)	17	196	213
	% within age group	8.0%	92.0%	100%
Adolescence (12-18 years)	Count (n)	11	84	95
	% within age group	11.6%	88.4%	100%
Total		42	573	615

The independent t-test did not reveal a statistically significant difference between the groups with and without adverse effects regarding the average dose of midazolam (t=-0.141; p=0.888) and ketamine (t=0.847; p=0.397) used. Therefore, in this study, the occurrence of adverse events was not dose-dependent. Children were sedated by a pediatric intensivist in 68.1% (n=419), by a general pediatrician in 26.7% (n=164), and by a pediatric resident (under the supervision of a specialist) in 2% (n=12). All medical staff had a pediatric advanced life support course. The physician responsible for sedation is unknown in 3.3% of cases (n=20). The Pearson chi-square test did not reveal a correlation between the occurrence of adverse events and the group of professionals who performed sedation (pediatrician from intensive care unit versus general pediatrician or pediatric resident) (Pearson chi-square test=0.123; p=0.725).

## Discussion

In this study, we describe our experience with the use of intravenous drugs for PSA. The most commonly used drugs were ketamine (99.2%) and midazolam (95.8%). Morphine and propofol were used in a small percentage of cases. The majority of patients received midazolam and ketamine in association at the dosages recommended in the literature.

The incidence of adverse events was low (8.1%) in our study, and all were resolved without the need for invasive interventions. There were no serious complications. Our findings are generally consistent with those previously reported in the literature; however, it should be noted that the frequency of adverse events varies between different published studies. In a retrospective study that included 243 patients, 215 sedated with ketamine, the rate of adverse effects was 9.8%, without any serious complications [9]. In another retrospective study, the group that received the combination of midazolam and ketamine (128 patients) during endoscopic exams demonstrated an adverse effects rate of 1.6%, with only one patient presenting hypoxemia [13]. Prospective studies carried out with sedation and analgesia in various procedures (with different therapeutic regimens, including the combination of midazolam and ketamine) have reported adverse effect rates ranging from 11.7% to 26% [14,15]. In our study, we observed a lower rate of adverse events compared to what has been described in some prospective studies [14-17]. This is a retrospective study and even though all patients were monitored, it is possible that minor transient adverse events without clinical significance may not have been registered. However, as described in other prospective studies in the literature, no serious complications were observed [14,16]. Severe complications requiring invasive measures are rare [18].

Our analysis revealed that the age groups with the highest incidence of adverse events were infants (0-12 months) and adolescents (12-18 years), and that respiratory complications were the most common, specifically transient oxygen desaturation (2%). The second most common adverse effect was vomiting. The reported adverse events are consistent with the findings of other studies. In a recent multicenter prospective study (2017) involving 6395 children, the most frequent adverse effects were hypoxemia (5.6%) and vomiting (5.2%) [15]. None of the patients required endotracheal intubation [15].

The majority of patients received midazolam and ketamine in association, a regimen that has been widely utilized in pediatric patients with favorable outcomes. In a prospective study of sedation for upper gastrointestinal endoscopy, which compared the combination of midazolam-ketamine (oral) with midazolam-placebo and midazolam-fentanyl (oral), it was concluded that the combination of oral ketamine and intravenous midazolam allowed for adequate and safe sedation [14]. In another study comparing midazolam-ketamine versus ketamine monotherapy, patients who underwent the combination did not present more adverse effects [19]. On the other hand, in a previous study, Roback et al. demonstrated that midazolam in combination with ketamine reduced the occurrence of vomiting but increased respiratory complications compared to ketamine monotherapy [20]. None of the patients with respiratory complications required endotracheal intubation and respiratory complications were more frequent in the ketamine-fentanyl group compared to the ketamine-midazolam group [20].

We also highlight the high number of sedations performed by general pediatricians (26.7%, n=164), although the majority of procedures were performed by pediatric intensive care physicians (68.1%, n=419). The occurrence of adverse events was not correlated with the physician responsible for the sedation (pediatric intensivist versus general pediatrician or pediatric resident). This is information that we did not find described in many studies (comparison between the physicians responsible for sedation). In our study, the performance of these procedures by general pediatricians (who typically have less experience with these drugs) has been shown to be safe. We emphasize the importance of all physicians having a pediatric advanced life support course and proper knowledge of the drugs used to safely perform these procedures.

The success of sedation depends on appropriate pre-sedation assessment, careful monitoring, and the presence of trained teams [16]. Children do not consistently respond to the recommended dose and sometimes a higher dose is necessary to achieve the desired effect. This is in line with our case series results. In our study, 33.4% of patients who underwent the combination of midazolam and ketamine required the administration of an additional bolus of at least one of the drugs to achieve the desired effect. In these cases, it is recommended to administer small repeated doses until adequate sedation is achieved. It is important to note that increasing the dose may result in the loss of airway reflexes and hypoventilation [1,16].

In our study, the occurrence of adverse events was not dose-dependent; however, other studies report different results. Bhatt et al. concluded that the use of a higher dose of ketamine was associated with a higher risk of vomiting and oxygen desaturation, but it was not associated with an increased occurrence of other serious adverse effects or significant interventions [15]. On the other hand, it is known that the respiratory depression effect of benzodiazepines such as midazolam is dose-dependent [4,6,7].

The low rate of adverse events without any serious complications supports the safety of intravenous sedation and analgesia in PED, particularly the combination of ketamine and midazolam. However, this study has some limitations. It is a retrospective study, conducted at a single center. Nonpharmacologic support for PSA was not registered but was not the objective of the present study. Nonpharmacologic support is protocolized and implemented by the majority of the staff, including the phase for intravenous access placement. These techniques help reduce preprocedural agitation, which permits an easier transition to sedation, may reduce the amount of medication required for sedation, and may decrease the rate of adverse events [21]. This study only includes intravenous drugs and does not include other routes of administration. On the other hand, despite the sample being representative (n=615), we believe that the real number of patients undergoing intravenous sedation and analgesia in the PED during the study period was higher than that recorded in the database. We hope that future multicenter prospective studies will contribute to the acquisition of further knowledge in this important area.

## Conclusions

The findings of this study indicate that the combination of midazolam and ketamine is a safe and effective method for sedation and analgesia in painful procedures, with a low rate of adverse events. Our results demonstrate that, with proper monitoring and trained professionals, intravenous sedoanalgesia enables the safe performance of painful procedures in the PED. These data are particularly relevant, as healthcare procedures frequently cause pain and discomfort in children and adolescents. We hope that our results will encourage pediatric services that have not yet adopted this practice to do so.
